# Evaluation of scoliosis screening using Moire topography in school children

**DOI:** 10.1186/1748-7161-10-S1-P9

**Published:** 2015-01-19

**Authors:** Akiko Misawa, Michio Hongo, Daisuke Kudo, Yoichi Shimada

**Affiliations:** 1 Akita Prefectural Center On Development and Disability, Japan; 2 Department of Orthopaedic Surgery, Akita University Graduate School of Medicine, Japan

## Objective

Screening of school children for scoliosis is an orthopedic examination that has a direct effect on the health of these children. Screening can be performed using various methods. Since 1983, in our prefecture, we have used Moire topography for screening. The purpose of this study was to evaluate the prevalence rate of scoliosis diagnosed by screening school children using Moire topography.

## Methods

Between 2005 and 2012, 33,840 students were screened: 22,897 elementary school boys and girls in the sixth grade and 10,943 junior high school girls in the second grade. Our screening program involved the use of Moire topography and radiography. This study was performed to determine the prevalence and distribution of scoliosis as well as the Cobb angle. When anomalous findings were observed, a roentgenographic examination of the entire spine was performed. The results were defined as follows: normal findings, a Cobb angle <10 degrees; non-reassuring, 10-14 degrees; regular examination, 15-24 degrees; and requires treatment, 25 degrees.

## Results

A total of 835 children (2.47%) were diagnosed using Moire topography. Radiographic examination revealed a Cobb angle of >10 degrees in 429 (61.3%) children. The overall prevalence rate of patients with Cobb angles >10 degrees was 1.27% (elementary school: 0.81%; junior high school: 2.22%). The findings revealed non-reassuring results in 0.46%, regular examination results in 0.61%, and the need for treatment in 0.20%.

Furthermore, the prevalence rate of patients with Cobb angles >15 degrees was 0.81%. The mean prevalence rate of Cobb angles >10 degrees during the last 4 years increased from 0.96% during the first 4 years to 2.51%, especially in elementary school children where the rate increased from 0.49% to 1.15%.

## Conclusions

Moire topography is more suited for screening scoliosis rather than for inspection and palpation due to limited oversight and the ability to screen a large number of school children. Recent data show that the prevalence rate in elementary school students increased; therefore, screening should be performed at an appropriate time to ensure early detection of scoliosis.

